# Asymmetrical putaminal atrophy in parkinsonism-predominant multiple system atrophy (MSA-P): A case report

**DOI:** 10.1016/j.radcr.2023.05.060

**Published:** 2023-06-20

**Authors:** Koichiro Mori, Akira Yagishita, Toshio Shimizu

**Affiliations:** a Department of Neuroradiology, Tokyo Metropolitan Neurological Hospital, Tokyo, Japan; b Department of Radiology, Tokyo Metropolitan Cancer and Infectious Diseases Center Komagome Hospital, 3-18-22 Honkomagome, Bunkyo-ku, Tokyo, 113-8677, Japan

**Keywords:** FLAIR, Putamen, Lateral medullary laminae, Asymmetrical parkinsonism

## Abstract

We encountered a case of multiple system atrophy parkinsonian subtype (MSA-P) with right-dominant parkinsonism in the early stage of the disease. Atrophy of the posterolateral putamen and iron deposition are the neuropathological hallmark of MSA-P. Coronal fluid-attenuated inversion-recovery (FLAIR) images showed atrophy and iron deposition in the left posterior putamen contralateral to the clinical dominant side in the early phase. Atrophy in the posterior putamen of patients with MSA-P was more clearly observed on coronal FLAIR images than on axial T2-weighted images. These findings reflected the pathological changes and might be a pathognomonic sign of MSA-P.

## Introduction

Multiple system atrophy (MSA) is a progressive neurodegenerative disease that clinically presents with various combinations of autonomic failure, parkinsonism, and cerebellar and pyramidal features. Pathologically, MSA is characterized by glial cytoplasmic inclusions and neuronal cell loss primarily in the olivopontocerebellar systems and striatum. MSA is classified as the parkinsonian subtype (MSA-P) when parkinsonism is the predominant symptom [1]. Pathologically, discoloration and atrophy of the posterolateral putamen are specific features of MSA, particularly MSA-P [2].

The clinical diagnosis of MSA has been made using the revised diagnostic criteria of Gilman et al. [3]. Recently, however, it has become clear that the clinical diagnosis of MSA is not always easy and that misdiagnosis is common [4], [5], [6]. This is because MSA presents a variety of clinical phenotypes, and there are many diseases similar to MSA, called mimics. Owing to the low sensitivity of the MSA diagnosis at first examination, many patients with early-stage MSA are excluded from clinical trials of disease-modifying drugs [7]. Therefore, the Movement Disorder Society Criteria for the Diagnosis of MSA [8] were published in 2022, and a new category, clinically established MSA, was introduced to achieve maximum specificity with acceptable sensitivity. The diagnosis of clinically established MSA requires brain MRI findings suggestive of MSA.

However, a highly specific diagnostic biomarker including MRI findings for MSA remains unestablished. We report a case of MSA-P in which atrophy and iron deposition of the posterior putamen were clearly observed on coronal FLAIR (fluid-attenuated inversion-recovery) images in the early stages of the disease.

## Case

**Fig. 1.**
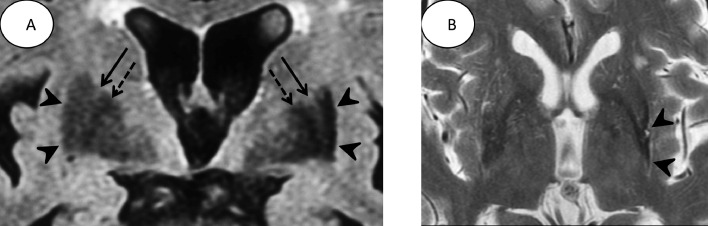
(A) Coronal fluid-attenuated inversion-recovery (FLAIR) image shows hypointensity in the left posterior putamen (arrowheads), which is clearly atrophic compared to that in the right putamen. The left lateral medullary lamina (arrow) is clearly visible and separated from the left atrophic putamen. The external globus pallidus shows hypointensity due to age-related iron deposition (dotted arrow). On the right side, the lateral medullary lamina (arrow) is also observed between the right putamen (arrowheads) and the external globus pallidus (dotted arrow). The right putamen is normal. (B) T2 weighted image (T2WI) reveals atrophy in the left putamen and hypointensity in the lateral side of the putamen (arrowheads) indicating iron deposition.

A 74-year-old man presented with tremor in his right upper limb and rigidity in both upper limbs that had persisted for nine months. There were no other neurological abnormalities. Coronal FLAIR images (Fig. 1) revealed atrophy and hypointensity in the left posterior putamen. The atrophied putamen showed hypointensity, indicating that it was clearly separated from the lateral medullary lamina. The right putamen was normal. Bilateral external globus pallidus showed hypointensity on coronal FLAIR images.

**Fig. 2.**
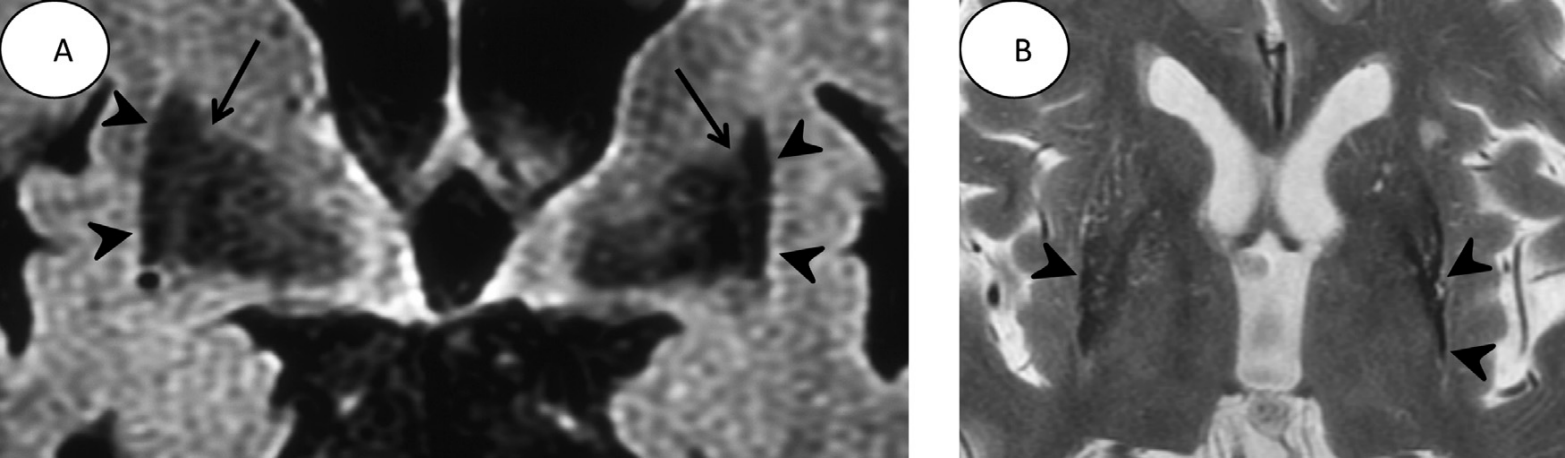
(A) In the coronal FLAIR image taken 2 years later, atrophy is observed in the right putamen (arrowheads) in addition to that in the left putamen (arrowheads). Moreover, the right posterior putamen has hypointensity. The lateral medullary lamine (arrows) can be identified on both sides. (B): T2WI shows progressive atrophy in the left putamen and hypointensity in the lateral side of the putamen (arrowheads). Hypointensity is also observed in the lateral side of the right putamen (arrowhead).

Two years later, the patient showed orthostatic hypotension and right-dominant cerebellar ataxia. Coronal FLAIR images (Fig. 2) demonstrated atrophy and hypointensity in the posterior bilateral putamens. Bilateral lateral medullary laminae were normal. Right-dominant cerebellar atrophy was also evident (not shown). In addition to parkinsonism, autonomic dysfunction defined as neurogenic orthostatic hypotension (≥20/10 mm Hg blood pressure drop within 3 minutes of standing) and cerebellar ataxia appeared. The patient met the diagnostic criteria for clinically established MSA [8].

## Discussion

We present a case of MSA-P with right-dominant parkinsonism in the early stages of the disease, which demonstrated left putaminal atrophy and iron deposition on MRI at 9 months from the onset. We believe that the following 2 points are important for the early diagnosis of MSA-P. First, MSA-P patients in the early stages present with parkinsonism with laterality. Second, MR images of MSA-P patients are characterized by atrophy and iron deposition in the putamen contralateral to the side of dominant parkinsonism, and these changes are clearly seen on coronal FLAIR images.

Atrophy of the posterolateral putamens and iron deposition are the neuropathological hallmark of MSA-P [9]. In our case, atrophy of the posterior putamen was clearly seen on coronal FLAIR images. MRI findings reflect pathological changes. Atrophy in the posterior putamen was better visualized on coronal images than on axial images because asymmetrical atrophy was directly observed on coronal images. Lateral medullary lamina and putamen in older patients were more clearly identified on coronal FLAIR images than on coronal T2-weighted images (T2WI). Hypointensity in the atrophic putamen on coronal FLAIR images indicates iron deposition as neurodegeneration. This finding differs from hypointensity in the external globus pallidus, which reflects iron deposition associated with age-related changes [10].

Fanciulli et al. [1] described the parkinsonism observed in MSA-P patients as occasionally asymmetrical. However, there have been reports of unilateral dominant parkinsonism in early stage MSA-P. Kikuchi et al. [11] presented a case of cogwheel rigidity in the right upper and lower limbs at the initial neurological examination. Foki et al. [12] also reported a patient with left hemi-parkinsonism. Our patient also exhibited right-dominant parkinsonism in the early stages of the disease. Moreover, in our hospital, almost all MSA-P patients had unilateral dominance of parkinsonism in the early stages.

In a report by Kikuchi [11], although a patient demonstrated right parkinsonism, there was no atrophy of the left putamen on axial T2WI. This might be because the evaluation was based on axial T2WI. If coronal FLAIR images had been taken, atrophy of the left posterior putamen might have been detected. Furthermore, atrophy in the putamen was not evident on axial T2WI in a study by Foki et al. [12].

As a limitation, this study reported only 1 case, but in our hospital, almost all MSA-P patients have unilateral dominance of parkinsonism in the early stages of the disease. Moreover, the first MR examinations are obtained within 2 years from the onset of parkinsonism, and coronal FLAIR images demonstrate atrophy and hypointensity in the posterior putamen contralateral to the clinically dominant side.

When coronal FLAIR images of patients with progressive asymmetrical parkinsonism obtained within 2 years from the onset show atrophy and iron deposition in the posterior putamen contralateral to the predominant side of parkinsonism, it might be a pathognomonic sign of MSA-P.

## Patient consent

The study participant had already died and informed consent for publication of this case report was obtained from this patient's wife.
